# *BRCA1* mutations attenuate super-enhancer function and chromatin looping in haploinsufficient human breast epithelial cells

**DOI:** 10.1186/s13058-019-1132-1

**Published:** 2019-04-17

**Authors:** Xiaowen Zhang, Yao Wang, Huai-Chin Chiang, Yuan-Pang Hsieh, Chang Lu, Ben Ho Park, Ismail Jatoi, Victor X. Jin, Yanfen Hu, Rong Li

**Affiliations:** 1 Department of Biochemistry & Molecular Medicine, School of Medicine & Health Sciences, The George Washington University, Washington, DC, 20037 USA; 2 Department of Molecular Medicine, University of Texas Health Science Center at San Antonio, San Antonio, TX 78229 USA; 3 Department of Chemical Engineering, Virginia Tech, Blacksburg, VA 24061 USA; 4 Vanderbilt-Ingram Cancer Center, Vanderbilt University Medical Center, Nashville, TN 37232 USA; 5 Department of Surgery, University of Texas Health Science Center at San Antonio, San Antonio, TX 78229 USA; 6 Department of Anatomy & Cell Biology, School of Medicine & Health Sciences, The George Washington University, Washington, DC, 20037 USA

**Keywords:** BRCA1, Transcription, Super-enhancer, Chromatin looping, Epigenetics, Breast epithelial cells

## Abstract

**Background:**

*BRCA1*-associated breast cancer originates from luminal progenitor cells. BRCA1 functions in multiple biological processes, including double-strand break repair, replication stress suppression, transcriptional regulation, and chromatin reorganization. While non-malignant cells carrying cancer-predisposing *BRCA1* mutations exhibit increased genomic instability, it remains unclear whether *BRCA1* haploinsufficiency affects transcription and chromatin dynamics in breast epithelial cells.

**Methods:**

H3K27ac-associated super-enhancers were compared in primary breast epithelial cells from *BRCA1* mutation carriers (*BRCA1*^*mut/+*^) and non-carriers (*BRCA1*^*+/+*^). Non-tumorigenic MCF10A breast epithelial cells with engineered *BRCA1* haploinsufficiency were used to confirm the H3K27ac changes. The impact of *BRCA1* mutations on enhancer function and enhancer-promoter looping was assessed in MCF10A cells.

**Results:**

Here, we show that primary mammary epithelial cells from women with *BRCA1* mutations display significant loss of H3K27ac-associated super-enhancers. These BRCA1-dependent super-enhancers are enriched with binding motifs for the GATA family. Non-tumorigenic *BRCA1*^*mut/+*^ MCF10A cells recapitulate the H3K27ac loss. Attenuated histone mark and enhancer activity in these *BRCA1*^*mut/+*^ MCF10A cells can be partially restored with wild-type BRCA1. Furthermore, chromatin conformation analysis demonstrates impaired enhancer-promoter looping in *BRCA1*^*mut/+*^ MCF10A cells.

**Conclusions:**

H3K27ac-associated super-enhancer loss is a previously unappreciated functional deficiency in ostensibly normal *BRCA1* mutation-carrying breast epithelium. Our findings offer new mechanistic insights into *BRCA1* mutation-associated transcriptional and epigenetic abnormality in breast epithelial cells and tissue/cell lineage-specific tumorigenesis.

**Electronic supplementary material:**

The online version of this article (10.1186/s13058-019-1132-1) contains supplementary material, which is available to authorized users.

## Background

Approximately 1 in 400 women in the USA carry germ-line *BRCA1* mutation (*BRCA1*^*mut/+*^) [1, 2]. These *BRCA1* mutation carriers have significantly higher risk of developing breast cancer compared to the general population, with an estimated cumulative risk of 65% by the age of 70 [3, 4]. While breast cancer screening could assist diagnosis at an early stage, it alone cannot reduce cancer risk [5]. The only effective risk-reducing options for women with *BRCA1* mutations are prophylactic mastectomy and oophorectomy, which can achieve 90% and 50% reduction in breast cancer risk, respectively [6–9]. However, due to the adverse physical and psychological effects, many at-risk women opt not to undergo these surgeries [10, 11]. Understanding functional deficiency that occurs prior to clinically evident cancer in precancerous *BRCA1*^*mut/+*^ breast epithelium is an important step towards developing alternative preventive strategies with higher precision and fewer side effects.

Mammary gland epithelium is composed of two lineages: luminal cells that surround the central lumen, and basal cells that are located adjacent to mammary stroma [12]. *BRCA1* haploinsufficiency leads to a luminal progenitor population deficiency in luminal cell differentiation [13–16]. Most *BRCA1*-associated breast tumors have a basal-like phenotype, with positive staining for the basal cell markers cytokeratin 5/6/14/17 and negative staining for the luminal cell markers estrogen receptor (ER) and progesterone receptor (PR) [17–20]. Of note, the basal breast cancer subtype is associated with poor clinical outcome [21]. However, *BRCA1*-associated basal-like breast tumors originate from luminal progenitor cells, namely, the cell of origin for *BRCA1*-associated tumors [13, 14, 16]. A major gap of knowledge in *BRCA1*-related cancer biology concerns the mechanism by which a single copy of *BRCA1* mutant allele leads to luminal differentiation deficiency and eventually basal-like tumors.

BRCA1 is best known for maintenance of genomic integrity through its functions in repair of double-strand DNA breaks via homologous recombination (HR) [22–24], regulation of cell cycle checkpoints [25, 26], and suppression of DNA replication stress [27]. When compared with their *BRCA1*^*+/+*^ counterparts, *BRCA1*^*mut/+*^ mammary epithelial cells function comparably in checkpoint regulation, yet exhibit haploinsufficiency in replication stress suppression and DNA repair [27–31]. While maintenance of genomic integrity is essential to BRCA1 tumor suppressor function, it alone does not easily explain the cell lineage-specific deficiency that occurs at early stages of tumorigenesis in *BRCA1* mutation carriers. BRCA1 is also implicated in transcriptional regulation and high-order chromatin reorganization [25, 32–37], processes that primarily dictate normal tissue development and cell differentiation. In support of this notion, multiple genome-wide studies show that BRCA1 preferentially binds to transcription start sites (TSSs) [38, 39]. Furthermore, our recent mouse genetic studies provide evidence for a functional crosstalk between BRCA1 and a bona fide transcription factor that regulates mammary luminal progenitor cell expansion and *BRCA1*-associated tumorigenesis [15, 40]. However, it remains unclear whether *BRCA1*^*mut/+*^ breast epithelial cells are haploinsufficient in regulation of transcription and chromatin dynamics.

Acetylated histones destabilize nucleosomes, increase chromatin accessibility for transcription factor binding, and ultimately facilitate gene expression [41, 42]. In particular, histone lysine 27 acetylation (H3K27ac) serves as a surrogate mark for active transcriptional enhancers [43]. Super-enhancers, which are large clusters of transcriptional enhancers, are bound by high levels of master regulatory transcription factors and co-factors [44, 45]. A high concentration of transcription factor binding renders rapid response of the corresponding target genes to various developmental cues [44, 46]. Super-enhancers, which are highly cell-type specific and enriched for H3K27ac, drive expression of genes that have essential roles in cell fate determination [45]. Notably, dysfunctional super-enhancers have been causally linked to pathogenesis including cancer [44, 45, 47–52]. Of note, BRCA1 interacts with CREB-binding protein (CBP) and p300, two structurally related histone acetyltransferases (HAT) that acetylate histones including H3K27 [32]. In addition, BRCA1 is found to interact with components of the histone deacetylase complex (HDAC) [35]. However, a potential role of BRCA1 in regulation of super-enhancer functions has not been investigated.

Here, we conducted whole-genome H3K27ac profiling of primary breast epithelial cells from *BRCA1* mutation carriers (*BRCA1*^*mut/+*^) and non-carriers (*BRCA1*^*+/+*^). Bioinformatics analysis indicates that heterozygous cancer-predisposing *BRCA1* mutation (*BRCA1*^*mut/+*^) dampens super-enhancer marks in primary human mammary epithelial cells (HMECs), in particular at those super-enhancers with GATA transcription factor binding. The effect of *BRCA1* mutations on super-enhancers was further corroborated using established non-tumorigenic breast epithelial cells engineered with a single copy of *BRCA1* mutant allele (*BRCA1*^*mut/+*^). Mechanistically, reduced H3K27ac levels in *BRCA1*^*mut/+*^ cells lead to impaired enhancer-promoter looping and decreased enhancer activity. Our work uncovers a previously unappreciated function of BRCA1 in super-enhancer regulation. The functional haploinsufficiency likely contributes to the cell lineage switch observed in early stages of *BRCA1*-associated breast tumorigenesis.

## Methods

### Breast tissue cohorts

Cancer-free breast tissues were procured from women either undergoing cosmetic reduction mammoplasty or prophylactic mastectomy, following protocols approved by the Institutional Review Board at the University of Texas Health Science Center at San Antonio. All donors signed written consent forms authorizing the use of the specimens.

### Primary epithelial cell isolation from human breast tissue

Fresh human breast tissue was processed as previously described [40]. In brief, tissue was digested in digestion buffer (DMEM/F-12 supplemented with 5% FBS, 0.1% BSA, 10 ng/mL epidermal growth factor, 10 ng/mL cholera toxin, 5 μg/mL insulin, 0.5 mg/mL hydrocortisone, 300 U/mL collagenase, and 100 U/mL hyaluronidase) on a 37 °C shaker overnight. Epithelium-enriched population was collected by centrifugation at 100 *g* for 3 min. Pellet was treated with 0.8% ammonium chloride to lyse red blood cells, followed by digestion with 0.05% trypsin-EDTA at 37 °C for 3 min. Cells were washed with washing buffer (HBSS supplemented with 2% FBS) and treated with dispase buffer (5 mg/mL dispase supplemented with 0.1 mg/mL DNase I) at 37 °C for 3 min. Single cells were obtained by passing through a 40-μm strainer.

### Chromatin immunoprecipitation (ChIP)

For H3K27ac/BRD4/CTCF ChIP, single cells were crosslinked with 1% formaldehyde at room temperature for 10 min, followed by incubation with 125 mM glycine for an additional 5 min. For MED1/BRCA1 ChIP, cells were crosslinked with 2 mM of disuccinimidyl glutarate (Thermo Fisher Scientific; 20593) at room temperature for 45 min, followed by further crosslinking with formaldehyde as described above. All following steps were carried out in buffers containing protease inhibitors in 4 °C until elution. Cells were pelleted by centrifugation at 1000*g* for 5 min, washed with PBS twice, then lysed in lysis buffer (5 mM HEPES, pH 7.9, 85 mM KCl, 0.5% Triton X-100) for 10 min. Nuclei were pelleted by centrifugation at 1600 *g* for 5 min and lysed in nuclei lysis buffer (50 mM Tris-HCl, pH 8.0, 10 mM EDTA, 1% SDS). Chromosomal DNA was sonicated using a Bioruptor Pico to obtain < 300-bp fragments. Ten percent of sonicated DNA was saved as input, and the rest was incubated with various antibodies overnight (H3K27ac: Abcam; ab4729. BRD4: Abcam; ab128874. CTCF: MilliporeSigma; 07-729. MED1: Bethyl Laboratories, Inc.; A300-793A. BRCA1: Bethyl Laboratories, Inc.; A300-000A). Dynabeads Protein A or G (Thermo Fisher Scientific; 10002D or 10003D) was added the following day and incubated for additional 4 h before washing. Washing was performed twice in TE sarcosyl buffer (50 mM Tris-HCl, pH 8.0, 2 mM EDTA, 0.2% sarcosyl), twice in TSE1 buffer (150 mM sodium chloride, 20 mM Tris-HCl pH 8.0, 2 mM EDTA, 0.1% SDS, 1% Triton X-100), twice in TSE2 buffer (500 mM sodium chloride, 20 mM Tris-HCl, pH 8.0, 2 mM EDTA, 0.1% SDS, 0.1% Triton X-100), twice in TSE3 buffer (250 mM lithium chloride, 10 mM Tris-HCl, pH 8.0, 1 mM EDTA, 1% sodium deoxycholate, 1% NP-40), and twice in TE buffer (50 mM Tris-HCl, pH 8.0, 2 mM EDTA). DNA was subsequently eluted from Dynabeads, reverse-crosslinked, and ethanol-precipitated. Locus-specific ChIP was assessed by PCR using primers as shown in Additional file 1: Table S2.

### ChIP-re-ChIP

For BRD4-H3K27ac ChIP-re-ChIP, samples were processed as described above prior to washing. BRD4-DNA-bound beads were washed three times in re-ChIP washing buffer (2 mM EDTA, 500 mM NaCl, 0.1% SDS, 1% NP40) and twice in TE buffer (50 mM Tris-HCl, pH 8.0, 2 mM EDTA). Samples were eluted in re-ChIP elution buffer (2% SDS, 15 mM DTT in TE buffer) by incubation at 37 °C for 30 min. After diluting 20 times with dilution buffer (16.7 mM Tris-HCl, pH 8.0, 0.01% SDS, 1% Triton X-100, 1.2 mM EDTA, 167 mM NaCl, 50 μg of BSA), samples were incubated with the re-ChIP antibody overnight, then processed as ChIP samples using the method described above.

### Library preparation and sequencing

H3K27ac chromatin immunoprecipitation with deep sequencing (ChIP-seq) libraries were constructed using a MicroPlex Library Preparation Kit (Diagenode; C05010011) following the manufacturer’s guide. After a total of 10 cycles of PCR amplification, libraries were purified using Agencourt AMPure XP System (Beckman Coulter; A63880). Quality and quantity of the libraries were measured by a Qubit dsDNA HS Assay Kit (Life Technologies; Q32851) using a Bioanalyser 2100. Libraries with different index sequences were pooled together and then sequenced with a single-end 50-bp module using an Illumina Hiseq 3000 system. De-multiplexing was performed by CASAVA to generate FASTQ files for each sample. Between 38 and 92 million unique mapped reads were obtained for each sample.

### Bioinformatics analysis of ChIP-seq

H3K27ac ChIP-seq was aligned to the human genome by BWA [53], and only unique mapped reads were saved. BELT [54], a bin-based peak calling algorism that applies a statistical method to control false discovery rate (FDR), was used to call peaks. Super-enhancers were identified using ROSE [45, 46]. Briefly, H3K27ac ChIP-seq peaks within 12.5 kb of one another were stitched together as enhancer clusters, then ranked and plotted based on the H3K27ac ChIP-seq signal. Stitched enhancer clusters that pass the inflection point in the distribution were designated as super-enhancers. HOMER [55] program was used for prediction of transcription factor binding sites. H3K27ac peaks located within super-enhancers were pooled together for motif search. Each super-enhancer was assigned a gene name based on closest proximity. ToppGene was used for Gene Ontology analysis [56].

### Cell culture

MCF10A with wild-type *BRCA1* or heterozygous *BRCA1* mutations were previously reported [29, 30] and cultured in DMEM/F12 (Thermo Fisher Scientific; 11330) supplemented with 5% of horse serum (Thermo Fisher Scientific; 16050), 20 ng/mL EGF (Gibco; PHG0311), 0.5 mg/mL hydrocortisone (Sigma; H0888), 100 ng/mL cholera toxin (Sigma; C8052), 10 μg/mL insulin (Sigma, I1882), and 1× penicillin-streptomycin (Thermo Fisher Scientific; 15070). Two days prior to the experiments, cells were trypsinized and 1.2 million cells were seeded in each 10-cm dish (MilliporeSigma; CLS3262).

### Chromatin conformation analysis (3C)

3C was performed following an established protocol with minor changes [57, 58]. In brief, MCF10A cells were trypsinized and counted. Ten million cells were used for each 3C condition. Cells were crosslinked with 1% formaldehyde at room temperature for 10 min, followed by 125 mM glycine at room temperature for 5 min. Cells were pelleted by centrifugation at 600 *g* at 4 °C for 5 min and re-suspended in pre-chilled lysis buffer (10 mM Tris-HCl, pH 8.0, 10 mM NaCl, 0.2% NP-40, 1 μg/mL leupeptin, 1 μg/mL aprotinin, 1 μg/mL pepstatin, and 1 mM PMSF). Samples were incubated on ice for 15 min and passed through a 21-G needle five times. Nuclei were pelleted by centrifugation at 2200*g* at 4 °C for 5 min, washed twice with NEB buffer 2.1, and re-suspended in NEB buffer 2.1. SDS was added to nuclei at a final concentration of 0.1%. Samples were incubated on a 65 °C shaker for 10 min then on ice immediately. Triton X-100 was added to quench SDS at a final concentration of 1%. Samples were incubated with 400 U of HindIII (New England Biolabs; R3104L) at 37 °C with rotation overnight. SDS was added to the samples the following day at a final concentration of 1.6%. Samples were incubated on a 65 °C shaker for 30 min and then transferred into 15-mL tubes with pre-chilled ligation buffer (1% Triton X-100, 0.8 mg BSA, 50 mM Tris-HCl, pH 8.0, 10 mM MgCl_2_, 10 mM DTT, and 2 mM ATP). After incubation with 300 U of T4 DNA ligase (Thermo Fisher Scientific; EL0011) at 16 °C for 4 h, samples were treated with 0.5 mg of proteinase K at 65 °C for 4 h, followed by an additional 0.5 mg of proteinase K treatment at 65 °C overnight. DNA was phenol-chloroform extracted the following day, diluted with distilled water, and ethanol-precipitated. Samples were treated with RNase A at 37 °C for 2 h, followed by phenol-chloroform extraction and ethanol precipitation. DNA was dissolved in TE at 4 °C overnight. Serially diluted 3C products were analyzed by PCR to determine linear range. 3C libraries within the linear range were analyzed by PCR using primers specific for the restriction fragments of interest. GAPDH was used for loading normalization. 3C primers are listed in the Additional file 1: Table S2.

## Results

### *BRCA1*^*mut/+*^ HMECs are associated with reduced super-enhancer mark

**Fig. 1:**
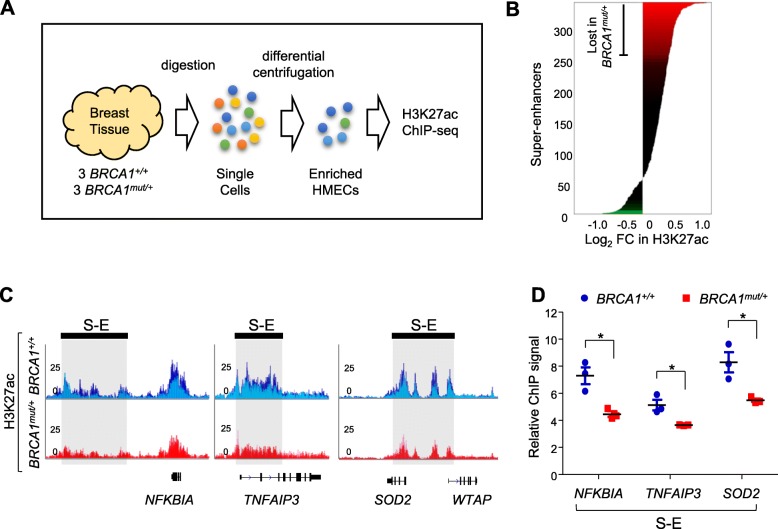
*BRCA1*^*mut/+*^ HMECs are associated with reduced super-enhancer mark. **a** Experimental design for H3K27ac ChIP-seq. Cancer-free human breast tissues (*BRCA1*^*+/+*^, *n* = 3; *BRCA1*^*mut/+*^, *n* = 3) were digested into single cells. Epithelial cell-enriched fractions were obtained through differential centrifugation. Primary HMECs were immediately crosslinked, and ChIP-seq was performed using an antibody specific for H3K27ac. **b** Differential analysis for super-enhancers. All genomic regions containing a super-enhancer in *BRCA1*^*+/+*^ or *BRCA1*^*mut/+*^ primary HMECs were ranked by log_2_ fold change in H3K27ac signal (*BRCA1*^*+/+*^ vs *BRCA1*^*mut/+*^). The *x-*axis shows log_2_ fold change in H3K27ac signals. Super-enhancers with log_2_ fold change ≥ 0.5 were colored red or green. **c** Track view of H3K27ac ChIP-seq density profile centered at three *BRCA1*^*mut/+*^-attenuated super-enhancers. Each track represents an overlay of three individual biological samples indicated by different colors. Locations of the super-enhancers are shaded and marked by black bars. **d** Confirmation of H3K27ac ChIP-seq by qPCR. Each dot represents one biological sample with *BRCA1*^*+/+*^ marked blue and *BRCA1*^*mut/+*^ marked red. *P < 0.05 by two-tailed *t* test. Error bars represent s.e.m

To compare super-enhancer landscapes in *BRCA1*^*+/+*^ and *BRCA1*^*mut/+*^ normal human breast epithelia, primary HMECs were isolated from fresh cancer-free breast tissues of *BRCA1* mutation carriers (*BRCA1*^*mut/+*^, *n* = 3) and non-carriers (*BRCA1*^*+/+*^, *n* = 3), who underwent prophylactic mastectomy and reduction mammoplasty, respectively. H3K27ac chromatin immunoprecipitation with deep sequencing (ChIP-seq) was performed [45, 59] (Fig. 1a), and super-enhancers were identified in each sample using established bioinformatics tool ROSE [45, 46]. A total of 343 super-enhancers were identified in *BRCA1*^*mut/+*^ and/or *BRCA1*^*+/+*^ breast epithelia, 268 of which were shared by *BRCA1*^*+/+*^ and *BRCA1*^*mut/+*^ HMECs (Additional file 2: Table S1, for an example see Additional file 3: Figure S1). H3K27ac intensity for 72 super-enhancers was lost or substantially attenuated in *BRCA1*^*mut/+*^ HMECs (Fig. 1b). Three representatives of such loci are shown in Fig. 1c, and the differential signals between *BRCA1*^*+/+*^ and *BRCA1*^*mut/+*^ were confirmed by locus-specific ChIP-qPCR (Fig. 1d). In contrast to the relatively large number of super-enhancers lost in *BRCA1* mutation carriers, only 3 super-enhancers were gained in the mutation-carrying samples (Fig. 1b, Additional file 2: Table S1). This result suggests a predominant role of wild-type (WT) BRCA1 in sustaining histone marks for super-enhancers in normal HMECs.

**Fig. 2:**
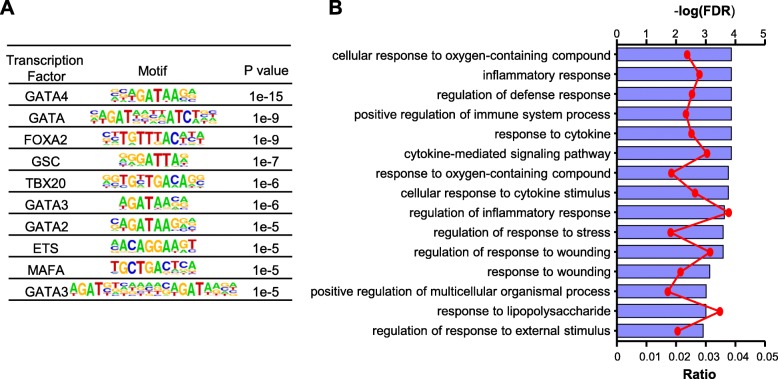
Motifs and pathways associated with super-enhancers attenuated in *BRCA1*^*mut/+*^ HMECs. **a** List of transcription factor binding motifs enriched in super-enhancers attenuated in *BRCA1*^*mut/+*^ HMECs. H3K27ac peaks within these super-enhancers were used for motif search. H3K27ac peaks within those super-enhancers shared by *BRCA1*^*+/+*^ and *BRCA1*^*mut/+*^ HMECs were used as background. The table is ranked by *P* value. **b** Gene Ontology (GO) analysis using genes associated with *BRCA1*^*mut/+*^-lost super-enhancers. ToppGene was used for GO analysis. Pathways were ranked by FDR-adjusted *P* value

To discern common features shared by those super-enhancers attenuated in *BRCA1*^*mut/+*^ HMECs, we used HOMER software suite [55] to identify transcription factor binding motifs that are enriched in these super-enhancers. We found that binding motifs for the GATA transcription factor family (GATA2, GATA3, and GATA4) are overrepresented in this group of super-enhancers (Fig. 2a). Using publicly available ENCODE ChIP-seq data performed in ER+ luminal breast cancer T47D cells [38], we found that 49 of the 72 super-enhancers missing in *BRCA1*^*mut/+*^ HMECs have GATA3-binding peaks. Given the well-established role of GATA3 in breast luminal epithelial fate determination [60–62], the reduced number of GATA-enriched super-enhancers could account for deficiency in luminal cell differentiation previously reported for *BRCA1*^*mut/+*^ HMECs [13–16]. We also used an established method to assign these super-enhancers to a total of 160 proximal potential target genes [44, 45, 63]. Gene ontology analyses show that this group of genes is enriched with those involved in various stress responses, including oxygen-containing compound, inflammation, and external stimulus (Fig. 2b). In summary, our genome-wide work of clinical samples indicates that *BRCA1* haploinsufficiency is associated with attenuated super-enhancers that have potential roles in lineage differentiation of human mammary epithelial cells.

### *BRCA1* haploinsufficient MCF10A cells recapitulate H3K27ac changes in primary *BRCA1*^*mut/+*^ HMECs

To corroborate the findings from primary HMECs, we used MCF10A cells that were genetically engineered to harbor a single allele of cancer-causing *BRCA1* mutation [29, 30]. MCF10A represents an immortalized yet non-tumorigenic human breast epithelial cell line with near normal diploidy. When cultured with extracellular matrix, MCF10A cells form acinar structures that recapitulate many aspects of mammary architecture in vivo [64, 65]. Previously published work has shown that *BRCA1*^*mut/+*^ MCF10A cells are prone to genomic instability, thus mimicking HMECs of *BRCA1* mutation carriers [29, 30].

**Fig. 3:**
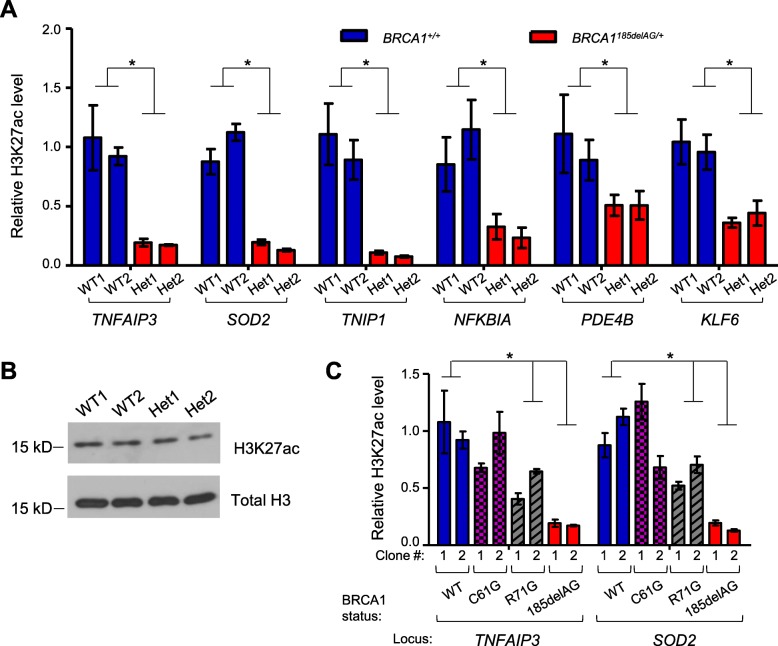
*BRCA1*^*185delAG/+*^ MCF10A cells recapitulate H3K27ac changes in *BRCA1*^*mut/+*^ HMECs. **a** Relative H3K27ac levels at six *BRCA1*^*mut/+*^-attenuated super-enhancers. The loci are named after the most proximal genes. The graph is an average of five independent experiments with two WT MCF10A clones (colored blue) and two *BRCA1*^*185delAG/+*^ MCF10A clones (colored red). **b** Western blot of H3K27ac in WT and *BRCA1*^*185delAG/+*^ MCF10A clones. Total histone H3 was used as the loading control. **c** Relative H3K27ac levels at *BRCA1*^*mut/+*^-attenuated super-enhancers. The graph is an average of three independent experiments with two WT MCF10A clones, two *BRCA1*^*C61G/+*^ clones, two *BRCA1*^*R71G/+*^ clones, and two *BRCA1*^*185delAG/+*^ clones. *P < 0.05 by two-tailed *t* test. Error bars represent s.e.m

We first examined H3K27ac marks in MCF10A clones carrying the most common pathogenic *BRCA1* mutation, 185delAG [30, 66]. To control for clonal variations, two independently targeted *BRCA1*^*185delAG/+*^ (Het1 and Het2, Fig. 3a) and two wild-type (WT1 and WT2) control MCF10A clones (*BRCA1*^*+/+*^, Fig. 3a) were evaluated by locus-specific H3K27ac ChIP. Six representative super-enhancers attenuated in primary *BRCA1*^*mut/+*^ HMECs were chosen for studies in the MCF10A-derived clones, each of which is named after the corresponding putative target genes. Similar to *BRCA1*^*mut/+*^ HMECs, both *BRCA1*^*185delAG/+*^ MCF10A clones had dramatically decreased H3K27ac levels compared to their WT counterparts (Fig. 3a). Of note, there was no significant difference in global H3K27ac levels between *BRCA1*^*185delAG/+*^ and WT cells (Fig. 3b and Additional file 3: Figure S2), suggesting that reduced H3K27ac signals in *BRCA1* mutant cells are likely due to BRCA1-dependent locus-specific changes in super-enhancer mark. We also assessed super-enhancer-associated H3K27ac status in MCF10A cells carrying a copy of two other common deleterious heterozygous *BRCA1* mutations [67, 68]. *BRCA1*^*R71G/+*^ clones exhibited significant reduction in H3K27ac intensity at two super-enhancers (*TNFAIP3* and *SOD2*) compared to WT clones (Fig. 3c), whereas *BRCA1*^*C61G/+*^ clones showed no appreciable H3K27ac changes (Fig. 3c). Taken together, *BRCA1* haploinsufficiency in both HMECs and established breast epithelial cells is associated with attenuated super-enhancer mark. However, severity of the deleterious effect likely depends on locations of the *BRCA1* cancer-predisposing mutations.

### Reduced H3K27ac level in *BRCA1* haploinsufficient cells attenuates action of enhancer-binding proteins and transcription of target genes

**Fig. 4:**
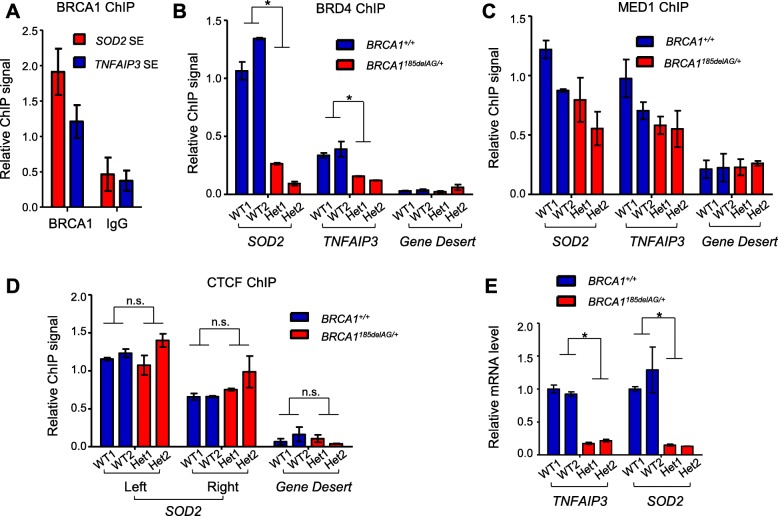
Reduced H3K27ac levels in *BRCA1*^*185delAG/+*^ MCF10A clones are associated with attenuated enhancer function and transcription. **a** Relative BRCA1 binding at *SOD2* and *TNFAIP3* super-enhancers in WT MCF10A cells. IgG binding was used as the control. The graph is an average of three independent experiments. **b** Relative BRD4 binding at *SOD2* and *TNFAIP3* super-enhancers. A desert zone with no genes or enhancers within 100 kb was used as the negative control. The graph is an average of three independent experiments with WT MCF10A and *BRCA1*^*185delAG/+*^ MCF10A cells. **c** Relative MED1 binding at *SOD2* and *TNFAIP3* super-enhancers. A desert zone with no genes or enhancers within 100 kb was used as the control. The graph is an average of three independent experiments. **d** Relative CTCF binding at *SOD2* super-enhancer. Two known CTCF peaks (left and right) located within *SOD2* super-enhancer were investigated. A desert zone with no CTCF binding was used as the control. The graph is an average of three independent experiments. **e** Relative mRNA expression of *TNFAIP3* and *SOD2*. The graph is an average of five independent experiments. *P < 0.05 by two-tailed *t* test. n.s. not significant by two-tailed *t* test. Error bars represent s.e.m

To gain more mechanistic insights into heterozygous *BRCA1* mutation-associated super-enhancer dysfunction, we used two representative super-enhancer loci *TNFAIP3* and *SOD2* because both promoters are associated with BRCA1 in various human cell lines as shown by publicly available BRCA1 ChIP-seq datasets [38, 39] (Additional file 3: Figure S3A and B). We first confirmed BRCA1 chromatin association with these two loci by ChIP in MCF10A cells (Fig. 4a). Next, we determined whether *BRCA1*^*185delAG/+*^ mutation status affects chromatin binding of bromodomain-containing protein 4 (BRD4), an epigenetic reader that binds to acetylated histone tails in active enhancer regions [69–71]. We observed a dramatic decrease in BRD4 chromatin binding at *TNFAIP3* and *SOD2* super-enhancers in *BRCA1*^*185delAG/+*^ MCF10A clones versus their WT counterparts (Fig. 4b). Of note, there was no significant difference in global BRD4 protein levels between *BRCA1*^*185delAG/+*^ and WT cells (Additional file 3: Figure S4). To directly test if BRD4 and H3K27ac are simultaneously associated with the same chromatin region, we performed BRD4-H3K27ac ChIP-re-ChIP. We found that BRD4 and H3K27ac co-occupy *TNFAIP3* and *SOD2* super-enhancers, and such co-occupancy was significantly decreased in *BRCA1*^*185delAG/+*^ cells compared to their WT counterparts (Additional file 3: Figure S5). These data strongly suggest that reduced H3K27ac level in *BRCA1* haploinsufficient cells directly attenuates epigenetic reading.

We also sought to determine whether *BRCA1* haploinsufficiency affects chromatin association of other enhancer-binding proteins at the aforementioned loci. MED1 is a subunit of the transcription coactivator Mediator complex that serves as a bridge to physically connect enhancers with their corresponding promoters and to transduce signals from various transcription factors to RNA polymerase II [72]. MED1 chromatin binding at the *BRCA1* mutation-affected super-enhancers showed a trend of decrease, albeit statistically insignificant, in *BRCA1*^*185delAG/+*^ MCF10A clones versus the WT control (Fig. 4c). We also assessed chromatin binding of CCCTC-binding factor (CTCF), which acts to shield undesired interactions between enhancers and promoters [73, 74]. Consistent with public datasets that indicate two CTCF binding peaks at the *SOD2* super-enhancer (Additional file 3: Figure S3A), we found that CTCF ChIP signals at these sites have similar intensity in WT control and *BRCA1*^*185delAG/+*^cells (Fig. 4d). Global CTCF levels were also similar in WT and mutant clones (Additional file 3: Figure S6). In accordance with reduced H3K27ac and BRD4 binding at these two super-enhancer loci, mRNA levels of *TNFAIP3* and *SOD2* were significantly diminished in *BRCA1*^*185delAG/+*^ MCF10A clones (Fig. 4e). Taken together, our data clearly suggest that haploinsufficient *BRCA1* mutation selectively impairs chromatin binding of enhancer-binding proteins and transcription of their downstream target genes.

**Fig. 5:**
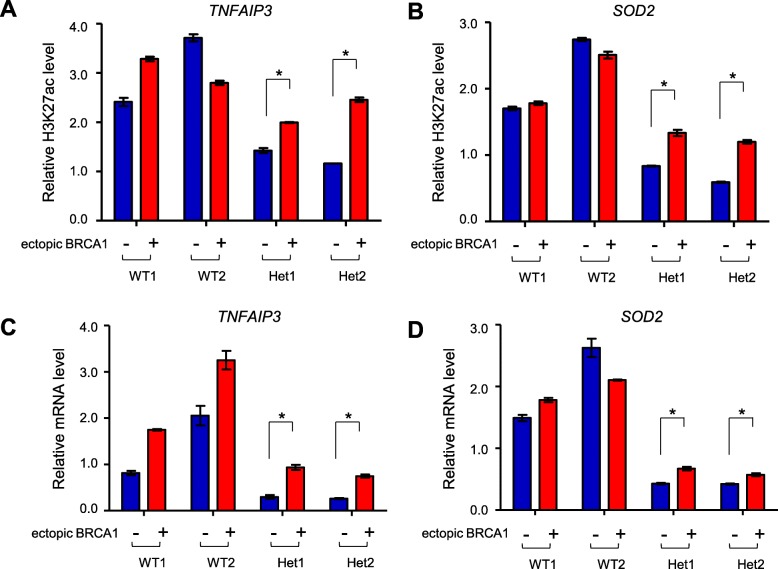
BRCA1 overexpression partially restores H3K27ac marks and transcription in *BRCA1*^*185delAG/+*^ MCF10A clones. **a**, **b** Relative H3K27ac levels at *TNFAIP3* (**a**) and *SOD2* (**b**) super-enhancers. The graph is an average of three independent experiments with control and *BRCA1* ectopic overexpression clones. **c**, **d** Relative mRNA expression of *TNFAIP3* (**c**) and *SOD2* (**d**). The graph is an average of three independent experiments. *P < 0.05 by two-tailed *t* test. Error bars represent s.e.m

*BRCA1* 185delAG contains a 2-nucleotide deletion in the second exon, leading to a frame shift and pre-mature translation termination immediately after the deletion. Indeed, *BRCA1*^*185delAG/+*^ MCF10A clones have lower WT BRCA1 level (Additional file 3: Figure S7). To ascertain that reduced H3K27ac intensity at various super-enhancers is indeed causally linked to lower WT BRCA1 expression in haploinsufficient cells, we ectopically expressed WT *BRCA1* in WT and mutant MCF10A cells. *BRCA1* overexpression did not change H3K27ac mark at *TNFAIP3* and *SOD2* super-enhancers in WT MCF10A clones, but substantially elevated H3K27ac levels at these two super-enhancer loci in *BRCA1*^*185delAG/+*^ clones (Fig. 5a, b). Concomitantly, mRNA levels of *TNFAIP3* and *SOD2* in mutant cells were also increased upon ectopic BRCA1 expression (Fig. 5c, d). These data strongly indicate that *BRCA1* haploinsufficiency directly influences H3K27ac intensity at these super-enhancers and their functionality in transcriptional activation.

### Impaired enhancer-promoter looping in *BRCA1* haploinsufficient cells

**Fig. 6:**
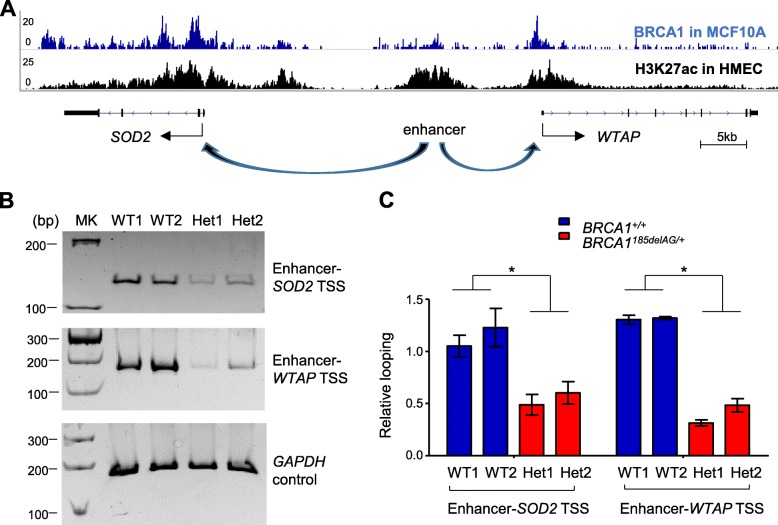
Impaired enhancer-promoter looping in *BRCA1*^*185delAG/+*^ MCF10A clones. **a** Schematic organization of *SOD2* super-enhancer region. *SOD2* TSS, *WTAP* TSS, and the enhancer regions to be investigated are marked. **b** 3C products of either WT MCF10A clones or *BRCA1*^*185delAG/+*^ MCF10A clones were analyzed by conventional PCR using primers detecting looping events between enhancer and TSSs. PCR products were run on a polyacrylamide gel and stained by ethidium bromide. 3C products were analyzed using GAPDH primers as the control. **c** Quantification of 3C-PCR products of **b**. *P < 0.05 by two-tailed *t* test. Error bars represent s.e.m

An important mechanism in transcriptional regulation in higher eukaryotes involves looping between distal enhancers and the corresponding proximal promoters [75–77]. To determine the impact of *BRCA1* haploinsufficiency on enhancer-promoter interactions, we carried out chromosome conformation capture (3C), the gold standard assay for examining long-distance chromatin looping [57, 78]. MCF10A cells were crosslinked to capture chromatin interactions, and restriction-digested DNA was ligated at a very low concentration that favors proximity ligation [57, 78]. We focused on the enhancer region that has the strongest H3K27ac signal in the *SOD2*-associated super-enhancer and interrogated its interactions with TSSs of two immediate flanking genes *SOD2* and *WTAP* (Fig. 6a). Compared to their WT counterparts, the two *BRCA1* mutant cell lines showed significantly decreased looping incidence between the enhancer and two flanking promoters (Fig. 6b, c). We conclude from these experiments that *BRCA1* haploinsufficiency affects long-distance chromatin interactions between transcription enhancers and promoters.

## Discussion

Combining studies of clinical samples and gene editing-generated isogenic cell lines, our work clearly demonstrates that a single copy of cancer-predisposing *BRCA1* mutation reduces super-enhancer mark and enhancer function in transcriptional activation. The causality of *BRCA1* haploinsufficiency and super-enhancer dysfunction is corroborated by partial rescuing of the phenotype with ectopic wild-type BRCA1. Collectively, our findings lend support to the notion that heterozygous *BRCA1* mutations are haploinsufficient for transcriptional regulation in non-tumorigenic breast epithelial cells prior to clinically evident cancer appearance.

BRCA1-associated breast tumors originate from luminal progenitor cells, yet they eventually become basal-like [13, 14, 16]. Deficient luminal cell maturation represents one of the earliest hallmarks of *BRCA1* mutation-carrying breast epithelium [14, 15]. Our data indicate that super-enhancers that are preferentially lost in *BRCA1*^*mut/+*^ HMECs are significantly enriched for GATA binding sites. Among the members of the evolutionally conserved GATA transcription factor family [79], GATA3 is known for its critical role in regulating luminal cell fate in the mammary gland [60, 61]. Notably, genetic ablation of mouse *Gata3* causes expansion of luminal progenitor cells and deficiency in luminal differentiation, which bears striking resemblance to *BRCA1*-deficient mammary epithelium [14, 15, 60]. Of note, it was reported that BRCA1 and GATA3 physically interact with each other to regulate gene expression [80]. Therefore, it is conceivable that BRCA1 promotes luminal differentiation by facilitating GATA3 transcriptional activity at the corresponding super-enhancers. We surmise that in breast epithelium of *BRCA1* mutation carriers, *BRCA1* haploinsufficiency could dampen GATA3 action in promoting luminal differentiation, which in turn drives the luminal-to-basal transition observed at early stages of *BRCA1*-associated breast tumorigenesis.

**Fig. 7:**
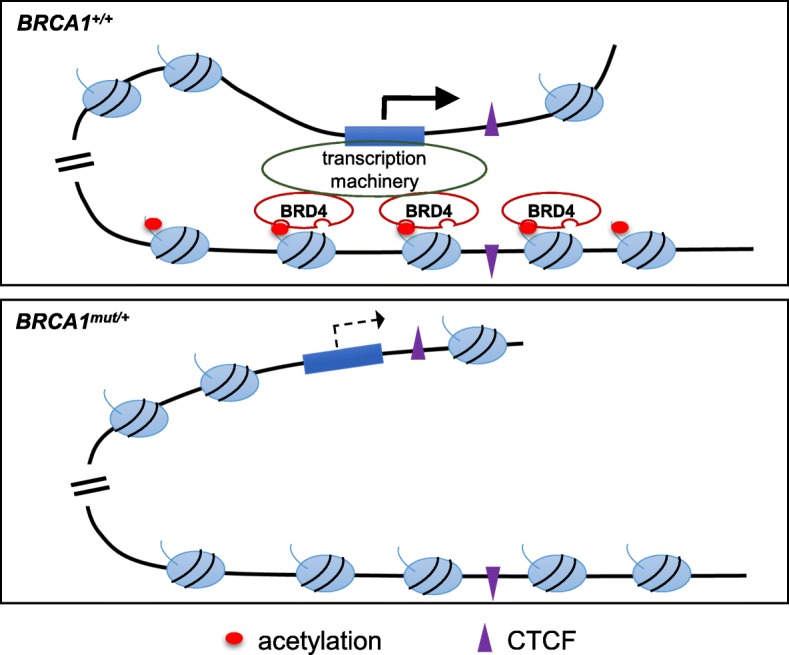
Model depicting *BRCA1* haploinsufficiency-associated chromatin looping deficiency. *BRCA1*^*mut/+*^ mammary epithelial cells display significant loss of H3K27ac, decreased association of BRD4, yet intact binding of CTCF, which ultimately results in impaired chromatin looping and transcriptional activation

BRD4, a member of the bromodomain and extraterminal (BET) family, is a reader of acetylated histones [69–71]. BRD4 regulates transcription through its interaction with the Mediator complex and positive transcription elongation factor b (P-TEFb) [81–84]. Genome-wide study found that BRD4 co-localizes with H3K27ac [46, 85], and its binding with acetylated histones is important for maintenance of higher-order chromatin structure [86]. Of note, super-enhancers are occupied by 16-fold more BRD4 than typical enhancers, and super-enhancer functions are preferentially affected by BRD4 inhibition [46]. On the other hand, CTCF binds to DNA in a methylation-sensitive manner [87, 88] and is primarily responsible for setting the boundaries of neighboring chromatin domains. Targeted degradation of CTCF leads to disruption of chromatin loops [89]. Our data clearly show that *BRCA1* haploinsufficiency (*BRCA1*^*mut/+*^) significantly weakens BRD4 chromatin binding and enhancer-promoter looping while keeping CTCF chromatin binding intact. This places BRCA1 action between CTCF and BRD4 in chromatin looping at the loci examined in our study. We propose a model whereby reduced H3K27ac in *BRCA1* haploinsufficient cells leads to decreased BRD4 recruitment, which in turn causes attenuated super-enhancer functions (Fig. 7). A role of BRCA1 in regulation of chromatin architecture is consistent with our earlier finding that, upon being tethered to chromatin, BRCA1 is capable of unfolding high-order chromatin structure [33].

While the biochemical basis for BRCA1 function in enhancer-promoter looping remains to be elucidated, several possible mechanisms are worth considering. First, BRCA1 could reinforce enhancer-promoter looping by recruiting HATs such as p300 and thus increasing H3K27ac density [32]. In a second scenario, BRCA1 is known to interact with RNA polymerase II (Pol II) and Pol II-pausing factor NELF-B/COBRA1 [33, 34]. In addition, BRD4 participates in regulation of transcription elongation [82–84]. In this regard, BRCA1 could strengthen enhancer-promoter looping through its interactions with factors involved in regulation of Pol II dynamics at the promoter-proximal region. In yet another alternative model, the potent ubiquitin E3 ligase activity of BRCA1/BARD1 heterodimeric complex has recently been implicated in histone H2A ubiquitination [90] and estrogen metabolism-related transcriptional regulation [91]. It is therefore conceivable that BRCA1/BARD1 E3 ligase-mediated chromatin modification could impact enhancer-promoter looping [92]. These possible mechanisms are not mutually exclusive, and further studies are warranted to shed more mechanistic light on three-dimensional chromatin reorganization in *BRCA1* mutation-carrying breast epithelium.

Our gene ontology analyses indicate that genes proximal to *BRCA1*-associated super-enhancers are enriched with those involved in cellular responses to various physiological cues including inflammation and stress. In particular, those involved in NF-κB and retinoic acid responses were identified in a previous study by Gardini et al. using an in vitro BRCA1 knock-down system in MCF10A cells [39]. Moreover, deregulated progesterone signaling [93, 94] and persistently active NF-κB pathway [95] were found in BRCA1-deficient mammary glands. However, because transcriptional enhancers do not always regulate expression of the most proximal genes, the functional link between BRCA1-affected super-enhancers and their neighboring genes used in our gene ontology analyses need to be experimentally validated. We are also cognizant of the limitation in using the immortalized cell line MCF10A to investigate BRCA1-regulated chromatin events and transcription, which obviously differs from primary breast epithelial cells in vivo. However, the fact that *BRCA1* haploinsufficiency displays a similar effect on the selected super-enhancers in clinical samples and MCF10A cells justifies the use of the cell line model for the in-depth mechanistic studies. Moreover, previously published findings using the same MCF10A-based cell culture model have provided physiologically relevant information concerning BRCA1 functions in regulation of epithelial differentiation and maintenance of genome stability [29, 30, 96, 97]. Given the various degrees of functional deficiency of *BRCA1* mutations in supporting super-enhancer activity, the in vitro system established in our study could serve as a convenient way of further exploring phenotype-genotype correlation for cancer-predisposing *BRCA1* mutations.

How germ-line *BRCA1* haploinsufficiency preferentially leads to tissue-specific cancer development remains a longstanding conundrum. Using haploinsufficient HMECs and cell line models, work from several laboratories supports the notion that genomic instability due to compromised BRCA1 activity in replication stress resolution and/or DNA repair contributes to BRCA1-associated tumorigenesis [27–31, 98]. Of note, Sedic et al. has shown that *BRCA1* haploinsufficiency-induced genomic instability occurs specifically in HMECs but not breast fibroblasts [28], which provides a molecular explanation for tissue-specificity of *BRCA1*-associated tumorigenesis. However, given the ubiquitous nature of DNA replication stress and DSB DNA repair, it is not clear whether genomic instability alone is sufficient to account for luminal-to-basal transition and subsequent cancer development in *BRCA1* mutation carriers. In this regard, mounting evidence suggests that BRCA1-mediated transcriptional regulation plays previously under-appreciated roles in tissue-specific tumor suppression. For example, the alternative NF-κB pathway is constitutively and preferentially active in BRCA1-deficient mammary luminal progenitor cells [95], the cell of origin for *BRCA1*-associated tumors. Furthermore, we recently showed that R-loops, transcription byproducts and DNA-RNA hybrids involved in genomic instability, preferentially accumulate in luminal epithelial cells but not in basal or stromal cells of *BRCA1* mutation-carrying breast tissue [40].

## Conclusion

Our current study provides a compelling molecular link between *BRCA1* haploinsufficiency and deficiency in super-enhancer functions and chromatin looping at a very early stage of *BRCA1* mutation-associated breast tumorigenesis. Conceptually, our findings strongly suggest that a direct role of BRCA1 in chromatin reorganization and transcriptional regulation contributes to its tissue-specific tumor suppressor function. A better understanding of the early molecular abnormalities in *BRCA1* mutation-carrying breast epithelium could potentially inform development of novel tools to more precisely prevent breast tumors in women with germ-line *BRCA1* mutations.

## Additional files

Additional file 1: **Table S2.** List of primer sequences used in this study. (XLSX 9 kb)

Additional file 2: **Table S1.** List of super-enhancers identified in this study. (XLSX 25 kb)

Additional file 3: **Figure S1.** Example of a super-enhancer shared by *BRCA1*^*+/+*^ and *BRCA1*^*mut/+*^ HMECs. Track view of H3K27ac ChIP-seq density profile centered at a *BRCA1*^*+/+*^ and *BRCA1*^*mut/+*^ HMECs-shared super-enhancer. Each track represents one biological sample with *BRCA1*^*+/+*^ colored blue and *BRCA1*^*mut/+*^ colored red. Locations of the super-enhancer are shaded and marked by a black bar, and TSS was marked by an arrow. **Figure S2.** Quantification of H3K27ac in *BRCA1*^*185delAG/+*^ MCF10A cells. Quantification of H3K27ac Western blot normalized by H3. Bar graph depicts the average of three independent experiments with WT MCF10A and *BRCA1*^*185delAG/+*^ MCF10A. Error bars represent s.e.m. n.s.: not significant by two-tailed *t* test. **Figure S3.** BRCA1 and CTCF ChIP-seq tracks. (A) Track view of published BRCA1 ChIP-seq [38, 39] and CTCF ChIP-seq [38] density profile centered on *SOD2* super-enhancer. Two CTCF peaks were marked. (B) Track view of existing BRCA1 ChIP-seq [38, 39] density profile centered on *TNFAIP3* super-enhancer. Locations of the super-enhancers are shaded and marked by solid bars, and TSSs are marked by arrows. Locations of the ChIP primers are marked in red. **Figure S4.** BRD4 level is not affected in *BRCA1*^*185delAG/+*^ MCF10A clones. (A) Western blot of BRD4 in WT and *BRCA1*^*185delAG/+*^ MCF10A clones. α-Tubulin was used as the loading control. (B) Quantification of BRD4 western blot normalized by α-Tubulin. Bar graph depicts the average of three independent experiments with WT MCF10A and *BRCA1*^*185delAG/+*^ MCF10A. **Figure S5.** Lower BRD4-H3K27ac co-occupancy in *BRCA1*^*185delAG/+*^ MCF10A clones. (A) Relative ChIP-re-ChIP signal at *SOD2* super-enhancer. The graph is an average of two independent experiments. (B) Relative ChIP-re-ChIP signal at *TNFAIP3* super-enhancer. The graph is an average of two independent experiments. *P < 0.05 by two-tailed *t* test. Error bars represent s.e.m. **Figure S6.** CTCF level is not affected in *BRCA1*^*185delAG/+*^ MCF10A clones. (A) Western blot of CTCF in WT and *BRCA1*^*185delAG/+*^ MCF10A clones. α-Tubulin was used as loading control. (B) Quantification of CTCF western blot normalized by α-Tubulin. Bar graph depicts the average of three independent experiments. Error bars represent s.e.m. n.s.: not significant by two-tailed t-test. **Figure S7.** Lower WT BRCA1 expression in *BRCA1*^*185delAG/+*^ MCF10A clones. Western blot of BRCA1 in WT and *BRCA1*^*185delAG/+*^ MCF10A clones. α-Tubulin was used as the loading control. (PPTX 170 kb)

## Abbreviations

3C: Chromosome conformation capture
BET: Bromodomain and extraterminal
BRD4: Bromodomain-containing protein 4
CBP: CREB-binding protein
ChIP-seq: Chromatin immunoprecipitation with deep sequencing
CTCF: CCCTC-binding factor
ER: Estrogen receptor
H3K27ac: Histone lysine 27 acetylation
HAT: Histone acetyltransferases
HDAC: Histone deacetylase complex
HMEC: Human mammary epithelial cell
HR: Homologous recombination
Pol II: RNA polymerase II
PR: Progesterone receptor
P-TEFb: Positive transcription elongation factor b
TSS: Transcription start site
WT: Wild-type
