# Supplementary material for: BRCA1 mutations attenuate super-enhancer function and chromatin looping in haploinsufficient human breast epithelial cells
Source: Breast Cancer Res. 2019 Apr 17;21:51. doi: 10.1186/s13058-019-1132-1 (PMC6472090; doi:10.1186/s13058-019-1132-1)
Supplement: Supplementary file 3 — Figure S1. Example of a super-enhancer shared by BRCA1+/+ and BRCA1mut/+ HMECs. Track view of H3K27ac ChIP-seq density profile centered at a BRCA1+/+ and BRCA1mut/+ HMECs-shared super-enhancer. Each track represents one biological sample with BRCA1+/+ colored blue and BRCA1mut/+ colored red. Locations of the super-enhancer are shaded and marked by a black bar, and TSS was marked by an arrow. Figure S2. Quantification of H3K27ac in BRCA1185delAG/+ MCF10A cells. Quantification of H3K27ac Western blot normalized by H3. Bar graph depicts the average of three independent experiments with WT MCF10A and BRCA1185delAG/+ MCF10A. Error bars represent s.e.m. n.s.: not significant by two-tailed t test. Figure S3. BRCA1 and CTCF ChIP-seq tracks. (A) Track view of published BRCA1 ChIP-seq [38, 39] and CTCF ChIP-seq [38] density profile centered on SOD2 super-enhancer. Two CTCF peaks were marked. (B) Track view of existing BRCA1 ChIP-seq [38, 39] density profile centered on TNFAIP3 super-enhancer. Locations of the super-enhancers are shaded and marked by solid bars, and TSSs are marked by arrows. Locations of the ChIP primers are marked in red. Figure S4. BRD4 level is not affected in BRCA1185delAG/+ MCF10A clones. (A) Western blot of BRD4 in WT and BRCA1185delAG/+ MCF10A clones. α-Tubulin was used as the loading control. (B) Quantification of BRD4 western blot normalized by α-Tubulin. Bar graph depicts the average of three independent experiments with WT MCF10A and BRCA1185delAG/+ MCF10A. Figure S5. Lower BRD4-H3K27ac co-occupancy in BRCA1185delAG/+ MCF10A clones. (A) Relative ChIP-re-ChIP signal at SOD2 super-enhancer. The graph is an average of two independent experiments. (B) Relative ChIP-re-ChIP signal at TNFAIP3 super-enhancer. The graph is an average of two independent experiments. *P < 0.05 by two-tailed t test. Error bars represent s.e.m. Figure S6. CTCF level is not affected in BRCA1185delAG/+ MCF10A clones. (A) Western blot of CTCF in WT and BRCA1185delAG/+ MCF10A clon [file 13058_2019_1132_MOESM3_ESM.pptx]

## Slide 1
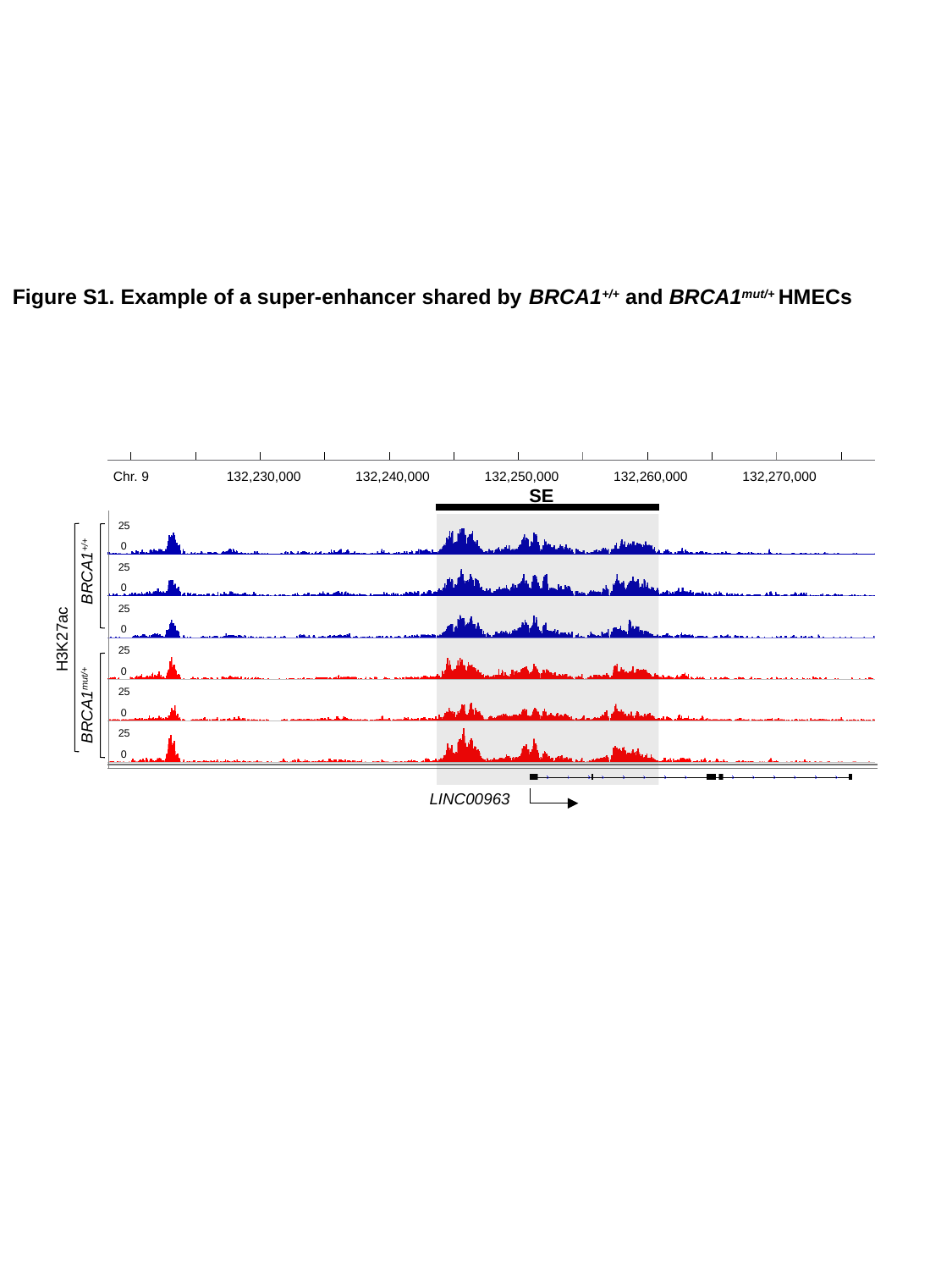

Figure S1. Example of a super-enhancer shared by BRCA1+/+ and BRCA1mut/+ HMECs
Chr. 9
132,230,000
132,240,000
132,250,000
132,260,000
132,270,000
SE
25
0
25
BRCA1+/+
0
25
0
H3K27ac
25
0
25
BRCA1mut/+
0
25
0
LINC00963

## Slide 2
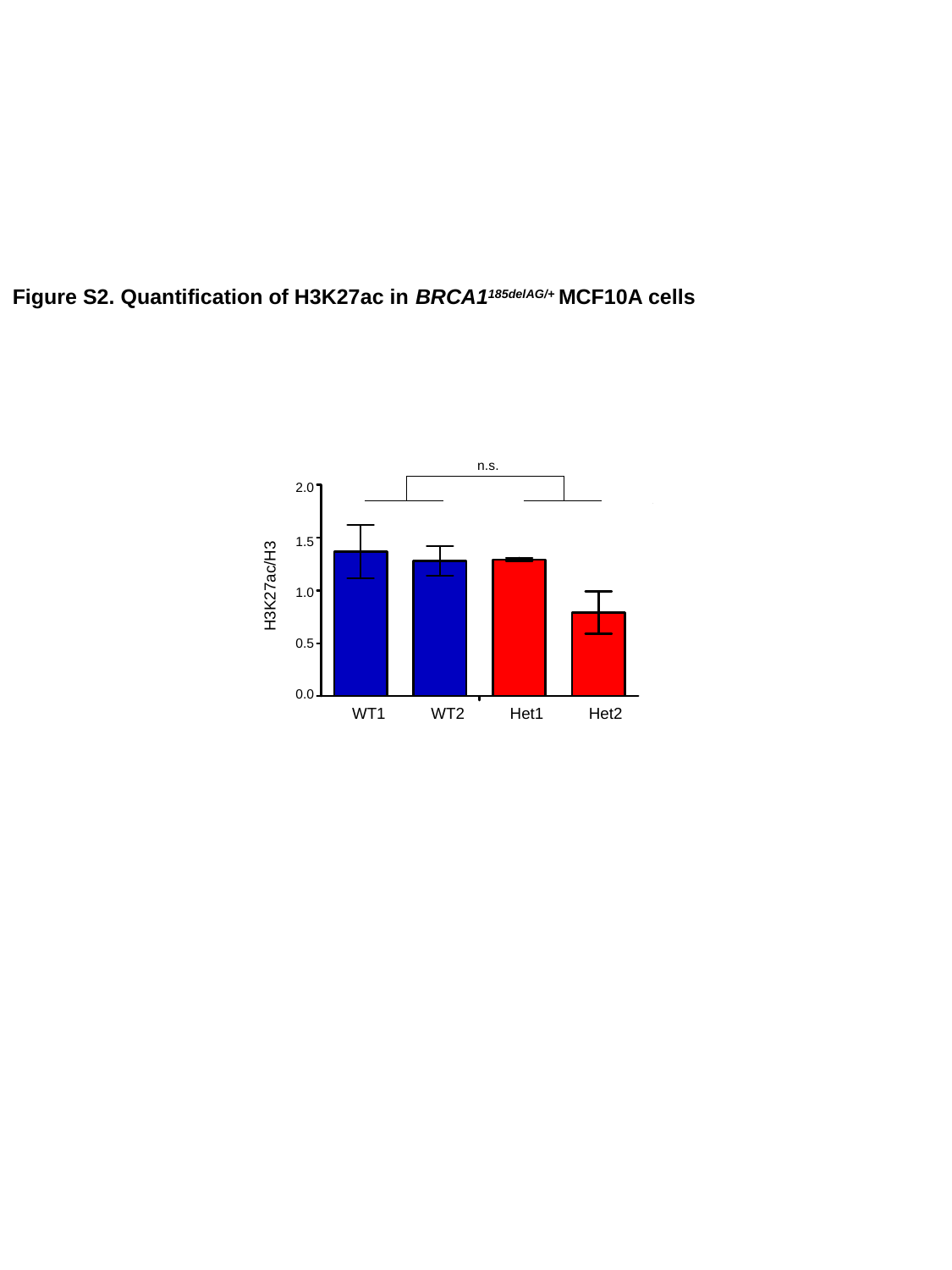

Figure S2. Quantification of H3K27ac in BRCA1185delAG/+ MCF10A cells
n.s.
2.0
1.5
H3K27ac/H3
1.0
0.5
0.0
WT1
WT2
Het1
Het2

## Slide 3
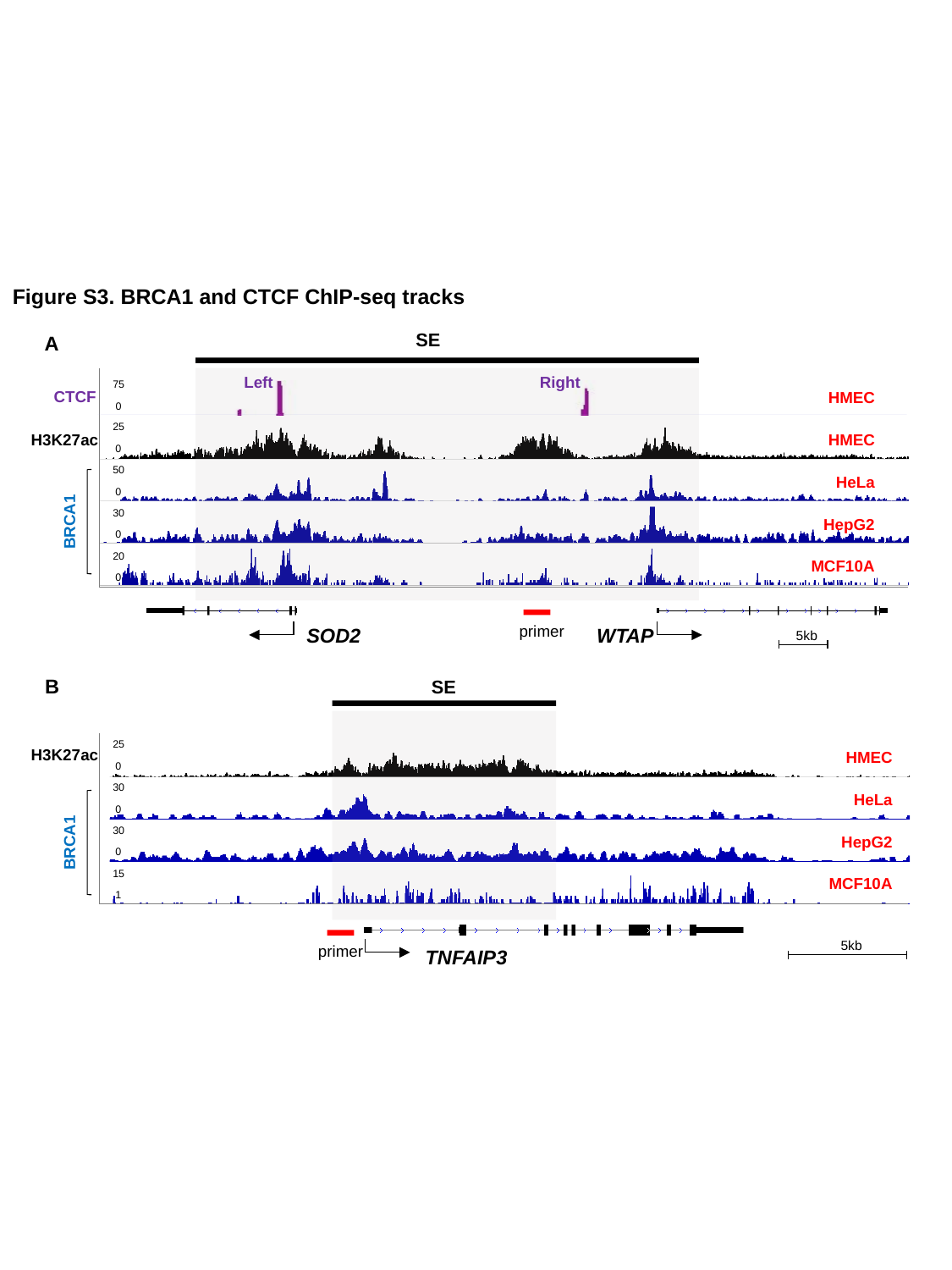

Figure S3. BRCA1 and CTCF ChIP-seq tracks
SE
75
CTCF
HMEC
0
25
HMEC
H3K27ac
0
50
HeLa
BRCA1
0
30
HepG2
0
20
MCF10A
0
primer
SOD2
WTAP
5kb
A
Left
Right
B
SE
25
H3K27ac
HMEC
0
30
HeLa
BRCA1
0
30
HepG2
0
15
MCF10A
1
5kb
primer
TNFAIP3

## Slide 4
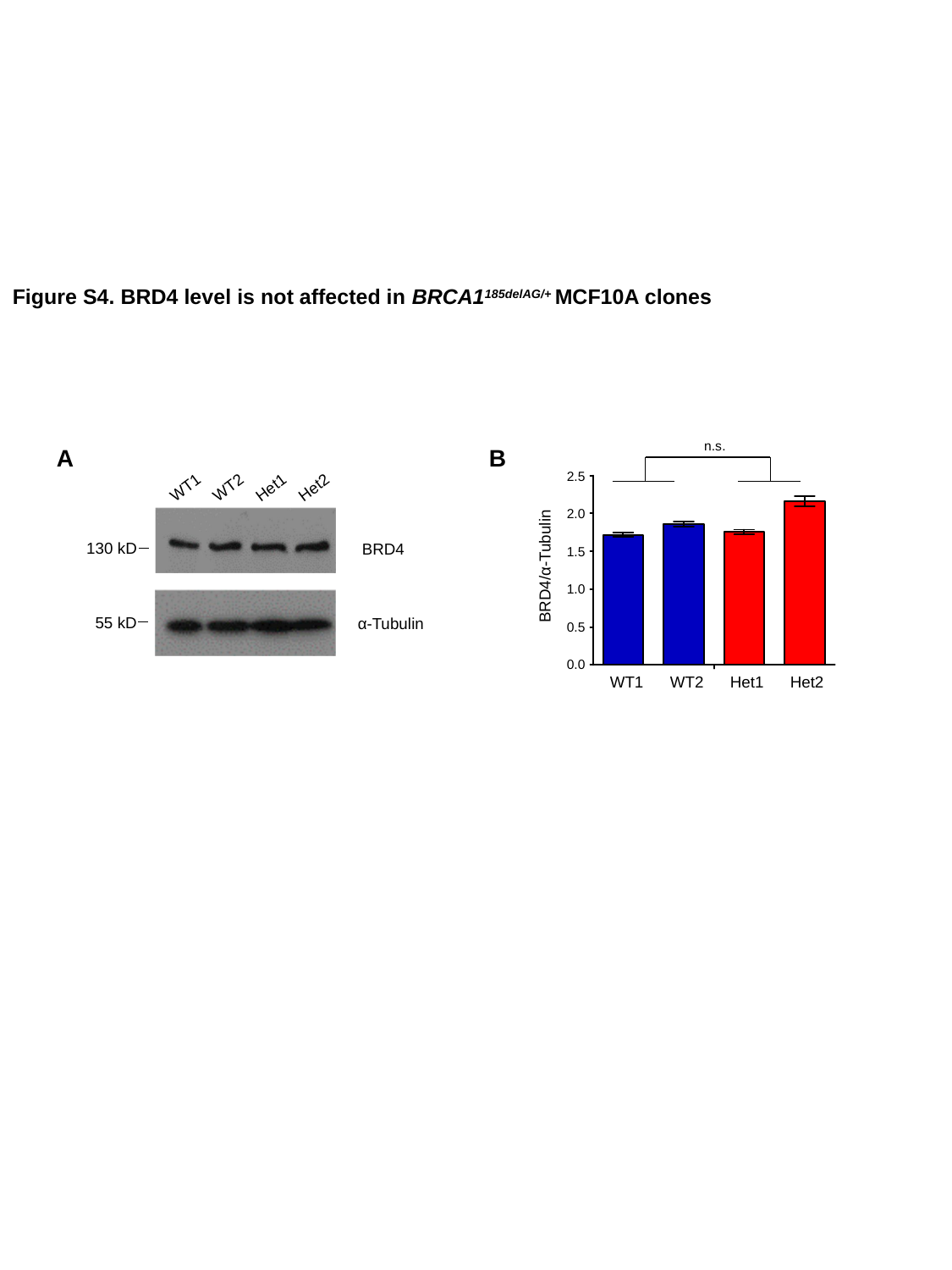

Figure S4. BRD4 level is not affected in BRCA1185delAG/+ MCF10A clones
n.s.
2.5
2.0
1.5
BRD4/α-Tubulin
1.0
0.5
0.0
WT1
WT2
Het1
Het2
A
B
Het1
Het2
WT1
WT2
130 kD
BRD4
55 kD
α-Tubulin

## Slide 5
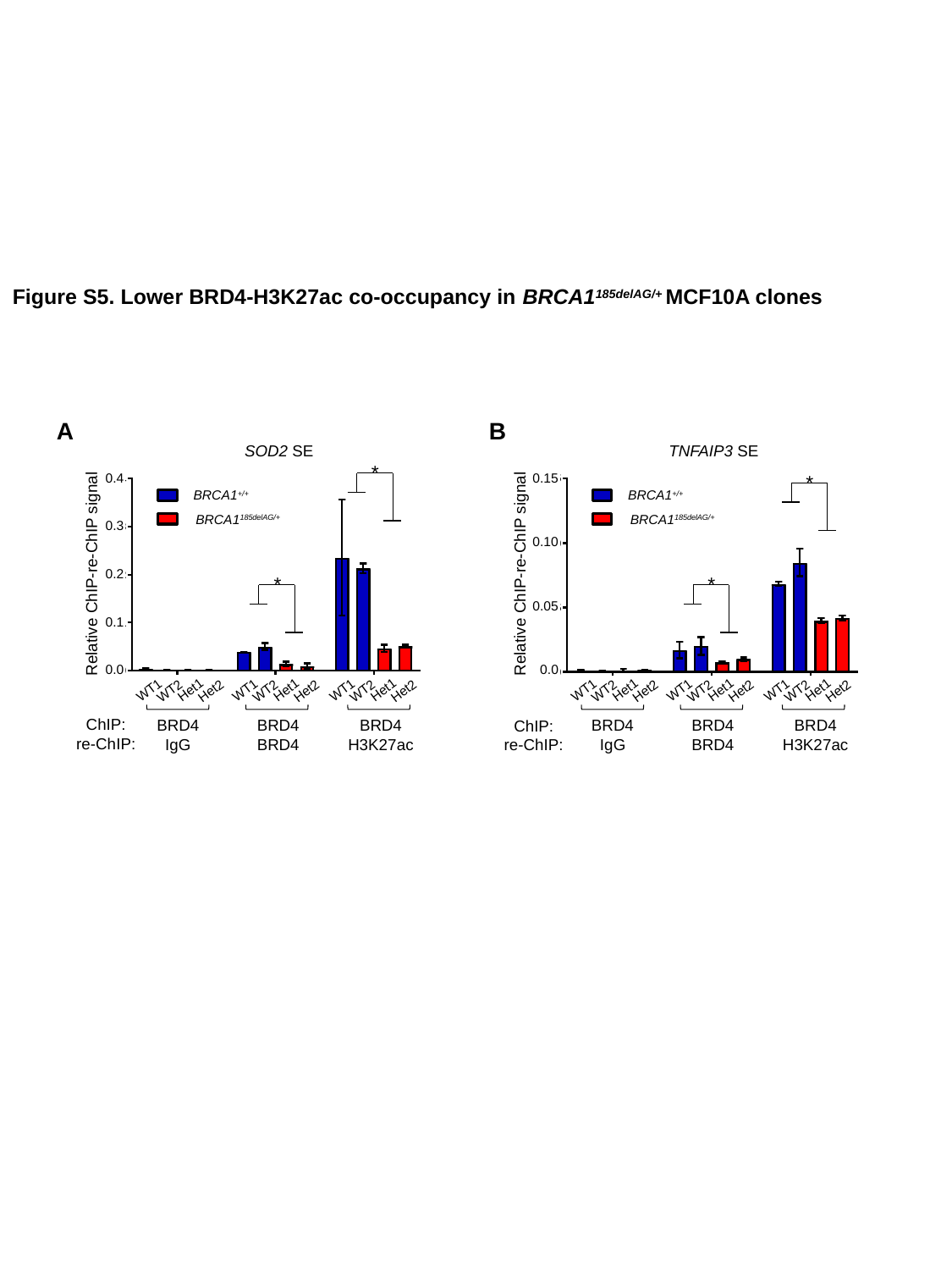

Figure S5. Lower BRD4-H3K27ac co-occupancy in BRCA1185delAG/+ MCF10A clones
A
B
SOD2 SE
*
0.4
BRCA1+/+
BRCA1185delAG/+
0.3
Relative ChIP-re-ChIP signal
0.2
*
0.1
0.0
Het1
Het1
Het1
WT1
WT1
WT1
WT2
WT2
WT2
Het2
Het2
Het2
BRD4
IgG
BRD4
BRD4
BRD4
H3K27ac
TNFAIP3 SE
0.15
*
BRCA1+/+
BRCA1185delAG/+
0.10
Relative ChIP-re-ChIP signal
*
0.05
0.0
Het1
Het1
Het1
WT1
WT1
WT1
WT2
WT2
WT2
Het2
Het2
Het2
BRD4
IgG
BRD4
BRD4
BRD4
H3K27ac
ChIP:
re-ChIP:
ChIP:
re-ChIP:

## Slide 6
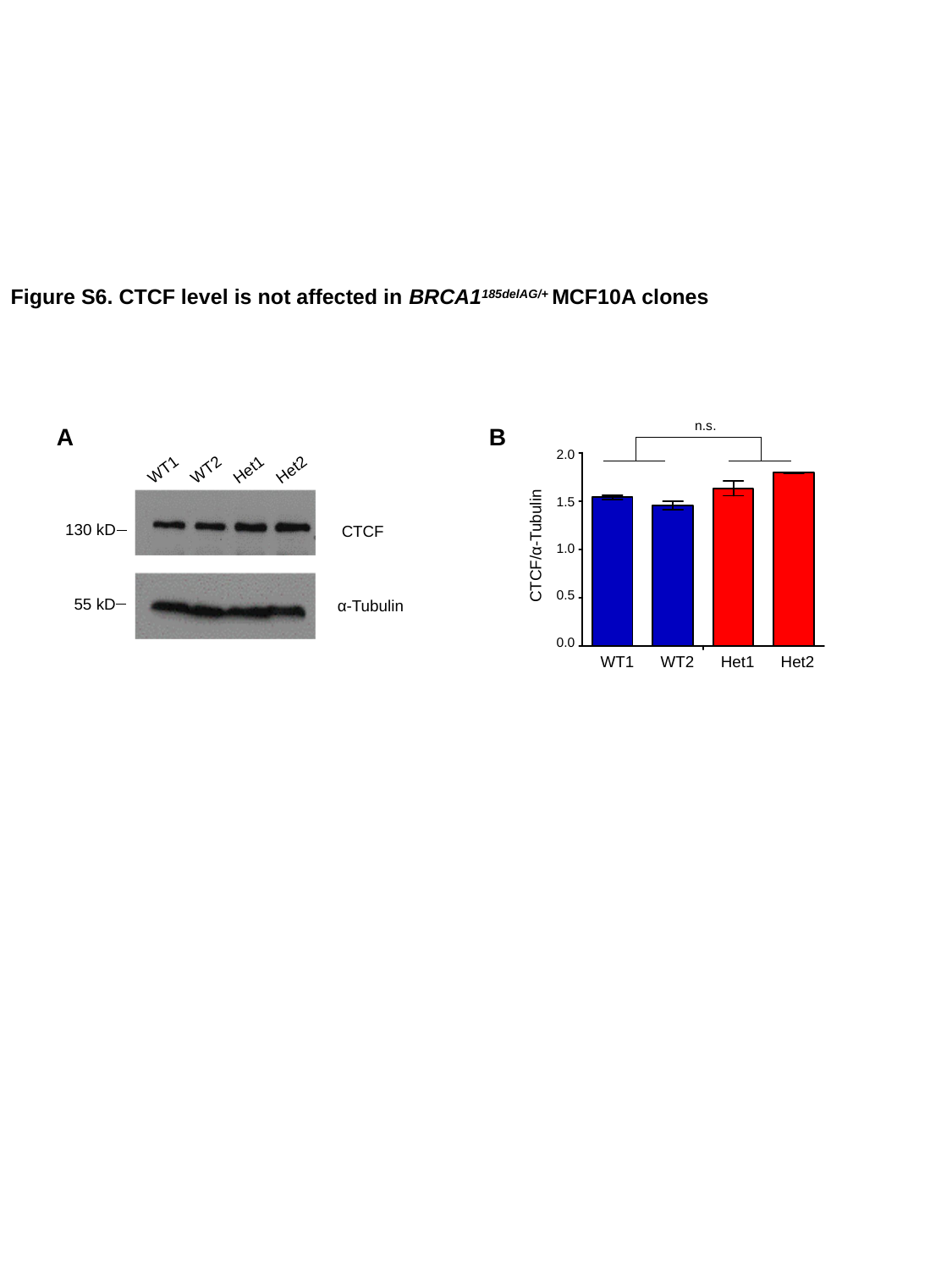

Figure S6. CTCF level is not affected in BRCA1185delAG/+ MCF10A clones
n.s.
2.0
1.5
CTCF/α-Tubulin
1.0
0.5
0.0
WT1
WT2
Het1
Het2
A
B
Het1
Het2
WT2
WT1
130 kD
CTCF
55 kD
α-Tubulin

## Slide 7
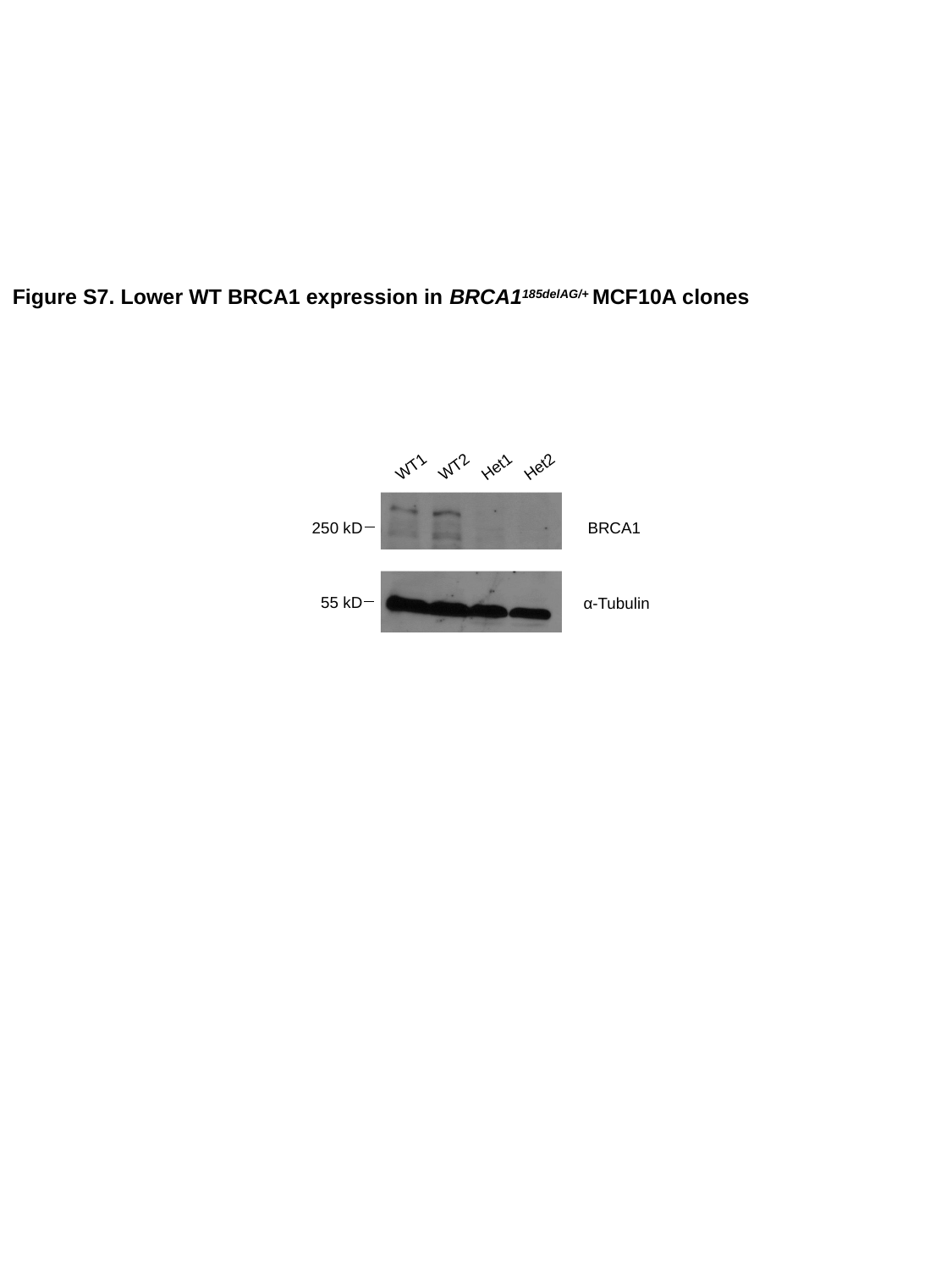

Figure S7. Lower WT BRCA1 expression in BRCA1185delAG/+ MCF10A clones
Het1
Het2
WT1
WT2
250 kD
BRCA1
55 kD
α-Tubulin
